# A Phase I, Dose Escalation, Single Dose Trial of Oral Attenuated *Salmonella typhimurium* Containing Human IL-2 in Patients With Metastatic Gastrointestinal Cancers

**DOI:** 10.1097/CJI.0000000000000325

**Published:** 2020-06-16

**Authors:** Thomas J. Gniadek, Lance Augustin, Janet Schottel, Arnold Leonard, Daniel Saltzman, Edward Greeno, Gerald Batist

**Affiliations:** * Department of Pathology, Northshore University Health System, Evanston, IL; Departments of † Biochemistry, Molecular Biology and Biophysics; ‡ Surgery, Division of Pediatric Surgery; ∥ Department of Medicine, Division of Hematology and Oncology, University of Minnesota, Minneapolis/St. Paul; § Salspera LLC, Oakdale, MN; ¶ McGill Centre for Translational Research in Cancer, Segal Cancer Centre, Jewish General Hospital, Montreal, QC, Canada

**Keywords:** microbial-based immunotherapy, Saltikva (Salmonella-IL2), phase I clinical trial

## Abstract

*Salmonella* has been shown to preferentially colonize solid tumors. It is known that toxicity limits the systemic administration of immunomodulatory cytokines that have a significant anticancer effect. Therefore, we tested a unique cancer treatment strategy comprised of oral delivery of Saltikva, an attenuated strain of *Salmonella typhimurium* that contain the human gene for interleukin-2. In preclinical experimentation, a significant antitumor effect without toxicity was observed. A dose escalation, single dose, Phase I trial was conducted. Dose escalation (10^5^ to 10^10^) while monitoring for dose limiting toxicity and response was performed. Flow cytometry was conducted to determine the immunologic effect. In total 22 patients were administered Saltikva. Eight patients did not complete the trial. No toxicity or adverse events were observed. There was no survival advantage. Flow cytometry demonstrated an increase in circulating natural killer (NK) cells and NK-T cells when comparing the prestudy period. The results of this phase I dose escalation study show that oral attenuated *S. typhimurium* containing the human interleukin-2 gene caused no significant toxicities up to doses of 10^10^ colony forming unit. There was no evidence of partial or complete response. All patients had progressive disease and eventually succumbed to their illness. Although no survival advantage was seen in this single dose study, the statistically significant increase in circulating NK and NK-T cell demonstrates an immunologic effect from this treatment regimen and suggest that a multiple dose study should be undertaken.

Despite great progress in oncologic therapy, cancer remains the second most common cause of death worldwide and 70% of those deaths occur in low and middle income countries, highlighting the desperate need for novel, affordable, and efficient therapeutic strategies.^1^

Because of their intrinsic immune-stimulating and cancer-targeting properties, microbial-based therapeutics are a promising potential treatment for cancer. Importantly, their appears far superior to cytotoxic chemotherapy and immunotherapy, however the results of animal studies and limited human trials have been mixed. Most studies administered bacterial therapy intravenously which can be associated with significant toxicity, initiated therapy long after the development of metastatic disease, or administered bacteria that did not contain any immunomodulatory proteins. More recent preclinical studies have shown that live bacterial therapy may be more effective at eliminating very early metastases or prevent the establishment of metastases altogether, a potential paradigm shift in management of cancer patients with high metastatic risk.^2^–^5^

Since *Salmonella typhimurium* naturally infects and colonizes solid tumors and stimulates a cellular immune response after infecting cells intracellularly, it represents an attractive anticancer therapeutic. To minimize the chance of bacterial-mediated complications, the χ4550 strain of *S. typhimurium* was created by deleting the cAMP and cAMP receptor genes (cya^−^/crp^−^). This strain was shown to be avirulent, but remained immunogenic. Furthermore, the strain was modified by deleting the chromosomal semialdehyde dehydrogenase (asd) gene required for cell wall synthesis, thereby ensuring uptake and maintenance of a plasmid (eg, pYA292) containing the asd gene without the need for antibiotic selection.^6^

In an attempt to further stimulate an antitumor cellular immune response, strain χ4550 was transformed with a biologically active, truncated human interleukin-2 (IL-2) gene-containing pYA292 plasmid, creating strain χ4550 (pIL-2) renamed, Salmonella-IL2. The presence of the asd gene in the plasmid ensures stable transfection and prevents reversion to a wild-type strain because if the bacteria were to lose the plasmid, it would not be able to construct a cell wall and die immediately.^7^

In vivo testing of Salmonella-IL2 in mice demonstrated that bacteria persisted in the liver and spleen for up to 6 weeks after oral administration of 2.5×10^8^ colony forming units (CFU) per mL in 0.25 mL (6.25×10^7^ CFU dose). Despite persistence of bacteria, the mice showed no ill effects. Furthermore, it has been shown that *S. typhimurium* accumulates within solid tumors at a ratio of 1000:1 to 10,000:1 over the normal liver and spleen indicating that *S. typhimurium* may be an extremely efficient method to deliver immune modulating proteins directly to the tumor microenvironment.^8^–^10^

Most promising are recent studies in dogs with metastatic osteosarcoma where 6 doses of orally administered Salmonella-IL2 was given every 3 weeks with standard of care amputation of the affected limb and every 3 week doxorubicin. This study demonstrated that Salmonella-IL2 was nontoxic in vivo. Furthermore, at 350 days, those dogs administered Salmonella-IL2 had a 40% disease free survival compared with 20% in historical controls. In addition, at 500 days, those dogs in the Salmonella-IL2 treated group had a 22% overall survival compared with none in the historical control group.^11^

This Phase I dose escalation study was designed to assess the safety of orally administered, attenuated Saltikva (Salmonella-IL2) to patients with metastatic carcinoma. In addition to adverse event monitoring, peripheral blood natural killer (NK) cell activity was assessed and progression was monitored. One limitation of this single dose study is that all patients had significant metastatic disease at enrollment, preventing an assessment of the efficacy of Saltikva therapy at preventing metastases or eliminating early metastases.

## MATERIALS AND METHODS

### Inclusion Criteria and Enrollment

**FIGURE 1:**
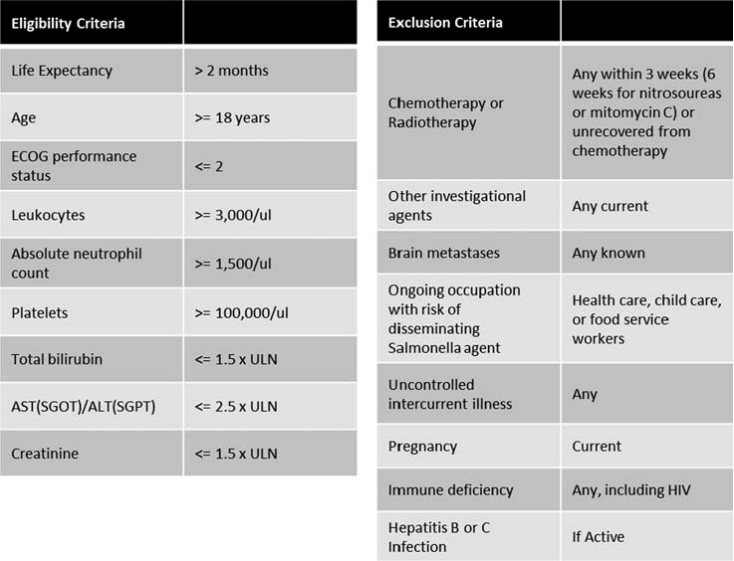
Eligibility and exclusion criteria. ALT indicates alanine aminotransferase; AST, aspartate aminotransferase; HIV, human immunodeficiency virus; SGOT, serum glutamic oxaloacetic transaminase; SGPT, serum glutamic pyruvic transaminase; ULN, upper limits of normal.

Patients with histologically confirmed solid tumors metastatic to the liver and no effective therapy available were eligible if they satisfied the inclusion criteria (Fig. 1), did not meet one of the exclusion criteria (Fig. 1), and were able to understand the written informed consent. Planned enrollment was up to 18 patients with 3 patients enrolled at each of 5 dose levels and an additional 3 patients at the maximum tolerated dose.

### Salmonella-IL2 Administration and Study Calendar

*Salmonella-IL2* was manufactured at the University of Minnesota Clinical Cell Therapy Facility, an accredited good manufacturing practice facility. Immediately before administration, each patient was given 30 mL of orally administered Maalox or Mylanta to neutralize gastric acid. A single dose of orally administered *Salmonella-IL2* was given during an outpatient visit. The dose was prepared by diluting a thawed glycerol stock in 30 mL saline. Immediately after administration, each patient was given 200 mL saline orally then took nothing orally for at least 1 hour.

**FIGURE 2:**
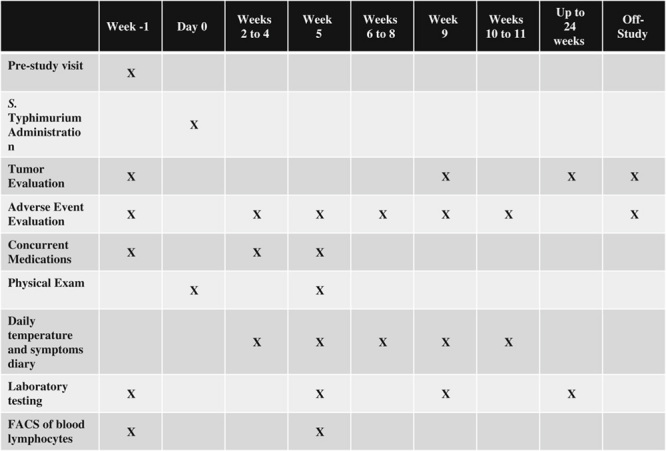
Study protocol. Imaging studies must be done <4 weeks before start of therapy. Prestudy visit includes informed consent as well as documenting height, demographics, medical history, and concurrent medications. In addition, prestudy laboratory testing includes beta-hcg (women of childbearing age) as well as HIV, hepatitis B, and hepatitis C testing. Physical examination includes physician medical examination, vital signs, weight, and performance status evaluation. Laboratory testing includes CBC with differential, serum chemistry [albumin, alkaline phosphatase, total bilirubin, bicarbonate, BUN, calcium, chloride, creatinine, glucose, LDH, phosphorus, potassium, total protein, SGOT (AST), SGPT (ALT), sodium]. Also, stool cultures performed on day 2 and 4 then weekly until negative for *Salmonella typhimurium* for 2 weeks. Blood cultures performed on day 2 and 4 then any time temperature >38.4°C. Off-study evaluations were included if documenting an adverse event or measurement of tumor size. ALT indicates alanine aminotransferase; AST, aspartate aminotransferase; BUN, blood urea nitrogen; CBC, complete blood count; FACS, fluorescent activated cell sorter; HIV, human immunodeficiency virus; LDH, Lactate dehydrogenase; SGOT, serum glutamic oxaloacetic transaminase; SGPT, serum glutamic pyruvic transaminase.

The planned dose escalation began with 10^5^ bacteria with an increase by one order of magnitude until a maximum dose of 10^10^ while monitoring for dose limiting toxicity. Description and grading scales for adverse event reporting were adopted from the revised National Cancer Institute Common Toxicity Criteria (CTC) version 3.0 guidelines. dose limiting toxicity was defined as sepsis syndrome, grade 4 vomiting or diarrhea, or other grade 3 toxicity. Close follow-up was planned for 11 weeks post administration with enrollment of 1 to 2 patients per month (Fig. 2).

### Supportive Care Guidelines

All vomiting or diarrhea were reported and managed as follows: For nausea, 10 mg po or 24 mg pr prochlorperazine (Compazine) was attempted, then other antiemetics (investigators discretion) were used if necessary. For loose stools up to 4 times per day above baseline, no treatment was necessary, however, loperamide or similar agents could be used for diarrhea of greater frequency. Intravenous hydration was used if necessary. Fever could be treated with antipyretics such as acetaminophen and an appropriate investigation was undertaken for the source of any fever >39.5°C. Any antibiotic therapy deemed necessary was given if an infectious source was identified, otherwise antibiotic therapy was not empirically started for fever.

### Safety Guidelines

To prevent transmission of the study agent to other individuals, all participants were instructed on good handwashing techniques and avoiding close contact with, or food preparation for infants, pregnant women, and immunocompromised individuals.

### Laboratory Testing

Clinical laboratory improvement amendments certified clinical laboratories were used for testing purposes, which included initial testing for pregnancy or infectious disease (Fig. 1B). Follow-up testing included serum chemistries and flow cytometric analysis of peripheral blood lymphocytes. Cell suspensions were stained and analyzed on an LSRII flow cytometer (BD Biosciences). Cells were gated and identified as follows: CD8 T-cells (CD8^+^), NK cells (CD8^−^, CD4^−^, CD49b^+^), Regulatory T-cells (CD25^+^, FoxP3^+^), monocytic MDSCs (CD45^+^, CD11b^+^, Ly6C^Hi^), granulocytic MDSCs (CD45^+^, CD11b^+^, Ly6G^Hi^). Antibody conjugates were purchased from BioLegend (San Diego, CA): αCD8/FITC (100705), αCD4/PerCP-Cy5.5 (100539), αCD49b/PE/Cy7 (108921), αCD45.2/BV510 (109837), αCD11b/BV650 (101239) and BD Pharmingen: αLy6C/PerCP/Cy5.5 (560525) and αLy6G/AF700 (561236) and eBioscience (Thermo Fisher Scientific, Waltham, MA): αFoxP3/Alexa Fluor 700 (56-5773-82). Patients receiving doses of 10^5^ through 10^9^ CFU had peripheral blood flow cytometry performed before administration and at 5 weeks post administration. Patients receiving 10^10^ CFU had additional peripheral blood flow cytometry performed at 2 and 3 weeks post administration, if possible.

### Measurement of Effect

Patients with measurable disease were assessed for response and progression using the Response Evaluation Criteria in Solid Tumors (RECIST) Committee criteria. Baseline imaging was planned as close as possible to date of administration, but no >4 weeks prior. If subsequent imaging showed complete or partial response, this was confirmed by repeat assessment within 4 weeks. Evidence of stable disease was confirmed by repeat imaging within 8 weeks.

## RESULTS

### Enrollment and Study Completion

**FIGURE 3:**
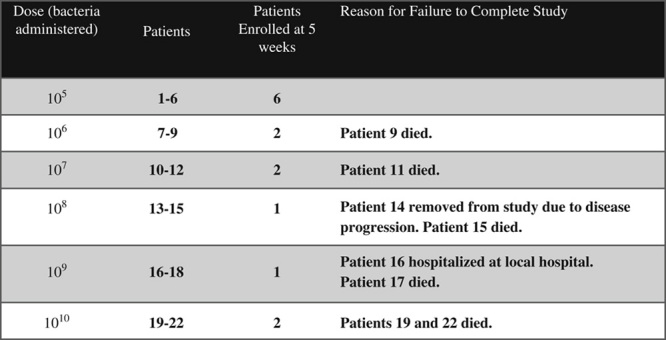
Enrollment and study completion.

**FIGURE 4:**
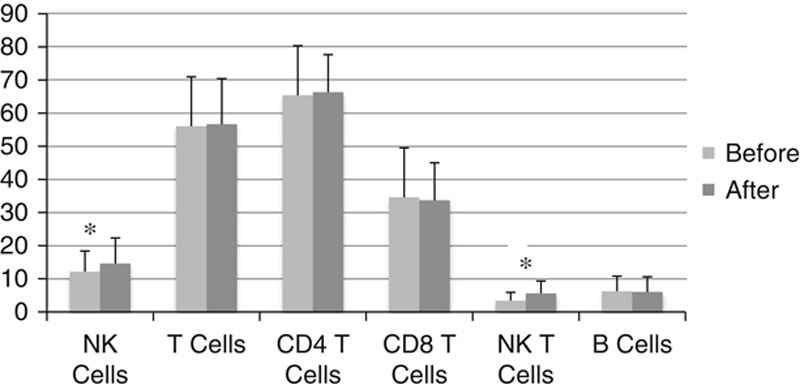
Peripheral blood lymphocyte flow cytometry: Effects of Salmonella-IL2 on blood lymphocyte population 5 weeks after oral administration demonstrating statistically significant elevation in NK and NK-T cell populations (*P<0.02). Nk indicates natural killer.

In total 22 patients were enrolled and administered *S. typhimurium* (Figs. 3, 4). Except for the lowest administered dose, at least 1 patient in each dose cohort died of cancer progression before 5 weeks follow-up (6 patients in total). One patient was removed from the study due to disease progression. One patient failed to have follow-up testing performed on week 5 due to hospitalization at a local hospital.

### Adverse Events

No adverse events were judged to be likely or definitely due to study enrollment. Two patients had adverse events possibly attributable to the study treatment: Patient 2 had anorexia, constipation, and hiccups at week 2. Patient 20 had fever at week 5. Both patients recovered. Three grade 3 adverse events were judged unlikely to be attributable to the study treatment: patient 4 had back pain at administration, patient 16 had back pain at week 6, and patient 19 had an elevated lymphocyte count.

### Blood and Stool Cultures

All blood and stool cultures were negative for the study organism.

### Serum Chemistries and White Blood Cell Differential

Overall, there was no statistically significant difference (*P*>0.05) between serum analyses tested at the prestudy visit compared with 5 weeks after administration (N=14). Comparing the low dose (10^5^) versus the high dose (10^10^) cohorts, there was a slight difference in the mean change in platelet count between prestudy and 5 weeks after administration (+58,500+/−86,720 vs. −40,667+/−356,978/mL, *P*=0.046), but there were no other statistically significant differences in the laboratory values tested.

### Off Study Antibiotic Usage

At 5 weeks post administration, patient 3 received nitrofurantoin 100 mg capsules and patient 10 received ciprofloxacin for an unrelated issue. Otherwise, no antibiotics were given during the course of the study.

### Peripheral Blood Lymphocyte Flow Cytometry

A comparison of samples taken preadministration with samples drawn 5 weeks post administration for all doses administered demonstrated a statistically significant increase in the percent peripheral blood NK cells (mean=12.1% vs. 14.6%, *P*=0.02) and NK-T cells (mean=3.4% vs. 5.6%, *P*=0.02), but no statistically significant difference in the other cell populations measured. The number of patients with 5-week post administration samples for each dose arm was 6 (10^5^ CFU), 2 (10^6^ CFU), 2 (10^7^ CFU), 1 (10^8^ CFU), 1 (10^9^ CFU), and 2 (10^10^ CFU).

### Response

There was no evidence of partial or complete response. All patients had progressive disease an eventually succumbed to their illness.

## DISCUSSION

The results of this Phase I dose escalation study show that a single oral dose of Saltikva caused no significant toxicities up to doses of 10^10^ CFU. The most commonly reported adverse events included pain in the abdomen or sides, weakness, and loose stools. Some patients experienced self-limiting diarrhea, however, no Salmonella was cultured in the stool. Peripheral blood chemistries and complete blood count with white cell differential showed no significant differences between the preadministration and post administration time periods. The cause of a slight difference in platelet count changes between the low and high dose cohorts is not readily apparent. Although no patients had a clinical response during the study period, all patients already had significant metastatic disease before enrollment.

Previous studies utilizing attenuated Salmonella or other bacteria have not been as promising as this one. A trial utilizing VNP20009 with patients with metastatic melanoma did not demonstrate survival efficacy or and revealed toxicity with intravenously administered bacteria.^4^ Similarly, a recent report of a Phase IIb study in patients with metastatic pancreas cancer were randomized to receive chemotherapy or a Listeria vaccine did not demonstrate a survival difference.^12^

The flow cytometric analysis of peripheral blood showed a statistically significant increase in the percentage circulating NK cells and NK-T cells when comparing the prestudy period to 5 weeks post administration. These finding were not dose dependent and were consistent with previous preclinical studies that showed an increase in systemic NK cell activation after administration of oral Salmonella-IL2, which may account for the effect of this therapy in decreasing the rate of metastatic osteosarcoma to the lung in the dog (an organ not typically associated with *S. typhimurium* infiltration).

Salmonella as a basis for cancer therapy are theoretically extremely attractive because of the unique propensity to colonize solid tumors at a ratio (1000 to 10,000:1) that is much higher than the “natural” target organs of Salmonella colonization during an infection, namely the liver and spleen.^2^,^13^ We have extensive preclinical data that demonstrated that orally administered Salmonella-IL2 also possesses this unique property. In fact, when various cancer cell lines were studied in vitro, we found that Salmonella-IL2 invaded hepatocytes most readily when compared with hepatoma, neuroblastoma, adenocarcinoma, and osteosarcoma. However, when examining the division efficiency, that is the ability to divide in a tumor once colonization occurred, osteosarcoma and neuroblastoma had the highest division efficiency.^14^ Furthermore, in vivo experimentation has demonstrated that orally administered Salmonella-IL2 can robustly colonize a variety of tumor types.^9^,^15^,^16^ Because Salmonella can colonize solid tumors, we maintain that Salmonella-IL2 can act as a “smart bomb” by invading and locally releasing IL-2 into the tumor microenvironment and avoid any systemic toxicity from the IL-2. In addition, Salmonella has been shown to demonstrate synergy with chemotherapy.^17^ Last, by orally administering Salmonella-IL2, we avoid any systemic toxicity related to the gram-negative bacteria itself because it was not delivered intravenously. Although it was proposed that patients undergo a tumor biopsy to ascertain if their tumors were colonized by Salmonella-IL2 in the Phase I trial, the Institutional Review Board asks us to remove this from our protocol, thus it could not be performed.

This study demonstrates safety and immunologic response to a single oral dose of Salmonella-IL2. Furthermore, the immunologic enhancement observed strongly suggest that further investigation take place with an expanded trial that would include a multiple dosing strategy in combination with standard of care chemotherapy for patients with metastatic and refractory cancers.

## References

[1] Noone AMHN Krapcho M Miller D SEER Cancer Statistics Review, 1975-2015. Bethesda, MD: National Cancer Institute; 2018.

[2] Forbes NS Coffin RS Deng L White paper on microbial anti-cancer therapy and prevention. J Immunother Cancer. 2018;6:78. 30081947 10.1186/s40425-018-0381-3 PMC6091193

[3] Flickinger JC Jr Rodeck U Snook AE Listeria monocytogenes as a vector for cancer immunotherapy: current understanding and progress. Vaccines (Basel). 2018;6:48–67. 10.3390/vaccines6030048 PMC6160973 30044426

[4] Toso JF Gill VJ Hwu P Phase I study of the intravenous administration of attenuated Salmonella typhimurium to patients with metastatic melanoma. J Clin Oncol. 2002;20:142–152. 11773163 10.1200/JCO.2002.20.1.142 PMC2064865

[5] Forbes NS Engineering the perfect (bacterial) cancer therapy. Nat Rev Cancer. 2010;10:785–794. 20944664 10.1038/nrc2934 PMC3756932

[6] Galan JE Nakayama K Curtiss R III Cloning and characterization of the asd gene of Salmonella typhimurium: use in stable maintenance of recombinant plasmids in Salmonella vaccine strains. Gene. 1990;94:29–35. 2227450 10.1016/0378-1119(90)90464-3

[7] Heise CP Saltzman DA Hasz DE Attenuated Salmonella typhimurium containing interleukin-2 decreases number of MC-38 hepatic metastases: a novel anti-tumor agent. Cancer Biother Radio. 1996;11:145–153. 10.1089/cbr.1996.11.145 10851531

[8] Saltzman DA Katsanis E Hasz DE Anti-tumor mechanisms of attenuated Salmonella typhimurium containing the gene for human interleukin-2. J Pediatr Surg. 1997;32:301–306. 9044141 10.1016/s0022-3468(97)90198-6

[9] Saltzman DA Cancer immunotherapy based on killing of Salmonella typhimurium infected tumor cells. Expert Opin Biol Ther. 2005;5:443–449. 15934824 10.1517/14712598.5.4.443

[10] Feltis BA Miller JS Sahar DA Liver and circulating NK1.1(+)CD3(-) cells are increased in infection with attenuated Salmonella typhimurium and are associated with reduced tumor in murine liver cancer. J Surg Res. 2002;107:101–107. 12384070 10.1006/jsre.2002.6428

[11] Fritz SE Henson MS Greengard E A phase I clinical study to evaluate safety of orally administered, genetically engineered Salmonella enterica serovar Typhimurium for canine osteosarcoma. Vet Med Sci. 2016;2:179–190. 29067193 10.1002/vms3.32 PMC5645873

[12] Le DT Picozzi VJ Ko AH Results from a Phase IIb, Randomized, Multicenter Study of GVAX Pancreas and CRS-207 Compared with Chemotherapy in Adults with Previously Treated Metastatic Pancreatic Adenocarcinoma (ECLISPE Study). Clin Cancer Res. 2019;25:5493–5502. 31126960 10.1158/1078-0432.CCR-18-2992 PMC7376746

[13] Pawelek JM Low KB Bermudes D Bacteria as tumour- targeting vectors. Lancet Oncol. 2003;4:548–556. 12965276 10.1016/s1470-2045(03)01194-x

[14] Soto LJ III Sorenson BS Nelson BW Preferential proliferation of attenuated Salmonella typhimurium with neuroblastoma. J Pediatr Surg. 2004;39:937–940. 15185229 10.1016/j.jpedsurg.2004.02.042

[15] Barnett SJ Soto LJ III Sorenson BS *Salmonella typhimurium* invades and decreases tumor burden in neuroblastoma. J Pediatr Surg. 2005;40:993–998. 15991184 10.1016/j.jpedsurg.2005.03.015

[16] Sorenson BS Banton KL Frykman NL Attenuated Salmonella typhimurium with IL-2 gene reduces pulmonary metastases in a model of osteosarcoma. Clin Orthop Relat Res. 2008;466:1285–1291. 18421536 10.1007/s11999-008-0243-2 PMC2384016

[17] Saltzman DA Augustin LB Leonard AS Low dose chemotherapy combined with attenuated salmonella significantly reduces tumor burden and is less toxic than high dose chemotherapy in an autochthonous murine model of breast cancer. Surgery. 2017;163:509–514. 29229318 10.1016/j.surg.2017.09.036

